# Avoidance Behavioral Difference in Acquisition and Extinction of Pain-Related Fear

**DOI:** 10.3389/fnbeh.2019.00236

**Published:** 2019-10-11

**Authors:** Yuki Nishi, Michihiro Osumi, Satoshi Nobusako, Kenta Takeda, Shu Morioka

**Affiliations:** ^1^Graduate School of Health Sciences, Kio University, Nara, Japan; ^2^Neurorehabilitation Research Center, Kio University, Nara, Japan; ^3^Department of Rehabilitation for the Movement Functions, Research Institute, National Rehabilitation Center for Persons with Disabilities, Saitama, Japan

**Keywords:** avoidance behavior, pain-related fear, fear response, extinction, protection from extinction

## Abstract

Fear of movement-related pain leads to two types of avoidance behavior: excessive avoidance and pain-inhibited movement. Excessive avoidance is an absence of movement by fear, and pain-inhibited movements involve a change in motor behavior for the purpose of protecting the painful part. Here, we sought to clarify the acquisition process and adaptation of fear for each avoidance behavior. Thirty-one female and 13 male (age 20.9 ± 2.1 years) subjects could decide persistent behaviors: approach with an intense pain stimulus, pain-inhibited movement with weak pain stimulus, or excessive avoidance with no pain in acquisition and test phases. In the subsequent extinction phase, the pain stimulus was omitted. Subjects were divided into an approach group (*n* = 24), a pain-inhibited movement group (*n* = 10), and an excessive avoidance group (*n* = 10) by cluster analysis. The response latencies in approach and pain-inhibited movement groups were not affected by conditioned pain. The subjects in the excessive avoidance group exhibited delayed response latencies, and their high-fear responses remained in the acquisition, test, and extinction phases. In addition, the excessive avoidance group showed high harm avoidance and high trait anxiety. This study demonstrated that differences in pain-related avoidance behaviors are affected by psychological traits. Pain-related excessive avoidance behavior indicated a maladaptive fear, but pain-inhibited movement did not.

## Introduction

Pain-related avoidance behaviors have short-term benefits which diminish movement-related pain and protect from further injury but sometimes become critical factors in the development or maintenance of chronic pain (Main and Watson, 1996; Pincus et al., 2006). Fear of movement-related pain leads to two types of avoidance behaviors: excessive avoidance and pain-inhibited movement. Excessive avoidance is a passive behavior such as complete disuse of the affected part and movement cessation due to pain (Hadistavropoulos et al., 1999; Tan et al., 2001; Bruehl and Chung, 2006; Garcia-Campayo et al., 2007). Pain-inhibited movements, such as changes in motor behavior undertaken to protect the affected part (Thomas and France, 2007, 2008), entail a spectrum of movement deviations and a decrease of the movement velocity in clinical practice (van Dieën et al., 2018). In addition, adaptation of pain-related fear has the key role of being the connection between avoidances and chronic pain (Vlaeyen and Linton, 2000). Adaptive fear induces avoidance in the only multisensory event that has been associated with nociceptive input, and thus, the individual’s activity level is maintained (Moseley and Vlaeyen, 2015). On the other hand, maladaptive fear leads to avoidance in the multisensory events that are not in fact dangerous and results in chronic pain (Leeuw et al., 2006; Karsdorp and Vlaeyen, 2009).

Previous studies about pain-related fear reported that pain-inducing movement resulted in fear of movement (i.e., delayed movement onset) and increased startle responses in the acquisition process (Meulders and Vlaeyen, 2012). The change of startle responses from acquisition phases to extinction is usually evaluated as the adaptation of fear. In addition, rewards that occur with movements are capable of modulating pain-related fear and avoidance (Claes et al., 2015, 2016). However, the relationship between the adaptations of fear and psychological traits has not been clarified. Patients with some chronic pain have been reported to exhibit specific personality traits such as high harm avoidance or high trait anxiety (Di Piero et al., 2001; Boz et al., 2007; Conrad et al., 2007; Mazza et al., 2009). Individuals with high harm avoidance or high trait anxiety have a tendency to respond intensely to previously established signals of aversive stimuli and to learn to passively avoid punishment (Cloninger et al., 1991).

In the present study, using prior research that clarified the relationship between pain-related fear and movements as our foundation, we attempted to determine whether pain-related fear induced by an original paradigm can be used to identify individuals who engage in pain-inhibited movement or excessive avoidance behaviors during free decision making, and we examined psychological traits related to each behavior. To prevent the vicious cycle of chronic pain, we contend that it is necessary to clarify the respective characteristics of individuals who acquire avoidance behaviors and to investigate whether those behaviors protect against the extinction of fear responses. We hypothesized that (1) the subjects who showed excessive avoidance of pain would have maladaptive fear [in other words, they show strong startle responses even if movement-related pain stimulation was stopped (the extinction phase)], and (2) these subjects would have high trait anxiety and harm avoidance because their personality traits urge passive avoidance (i.e., excessive avoidance).

## Materials and Methods

### Participants

Forty-four healthy volunteers (31 women and 13 men; mean ± SD age: 20.9 ± 2.1 years) were recruited at Kio University. The study protocol conformed to the Declaration of Helsinki. Before participating, each subject provided written informed consent. This study was approved by the Ethics Committee of Kio University Health Science Graduate School (approval no. H27-27).

The exclusion criteria were neurological disease, any current or past psychiatric disorder including clinical depression and chronic pain, hearing problems, painful wrist/hand or related problems, a cardiac pacemaker or the presence of any other electronic medical device, and the presence of any other severe medical conditions.

We calculated the necessary sample size, which we estimated by *a priori* power analysis with reference to the confirmation experiment (see Supplementary Figures S1, S2). The effect size was 0.38, and the correlation among repeated measures was 0.4. The total required sample size was 39 subjects.

### Movement Paradigm

We created a paradigm that can measure continuous pain-related avoidance behavior in a fixed time frame. We conducted the confirmation experiment in advance to determine whether pain-related fear induced by an original paradigm can be used to identify individuals who engage in pain-related avoidance behavior during free decision making (see Supplementary Figures S1, S2). The experiment was programmed using the software program LabVIEW (National Instruments, Austin, TX, United States).

**FIGURE 1:**
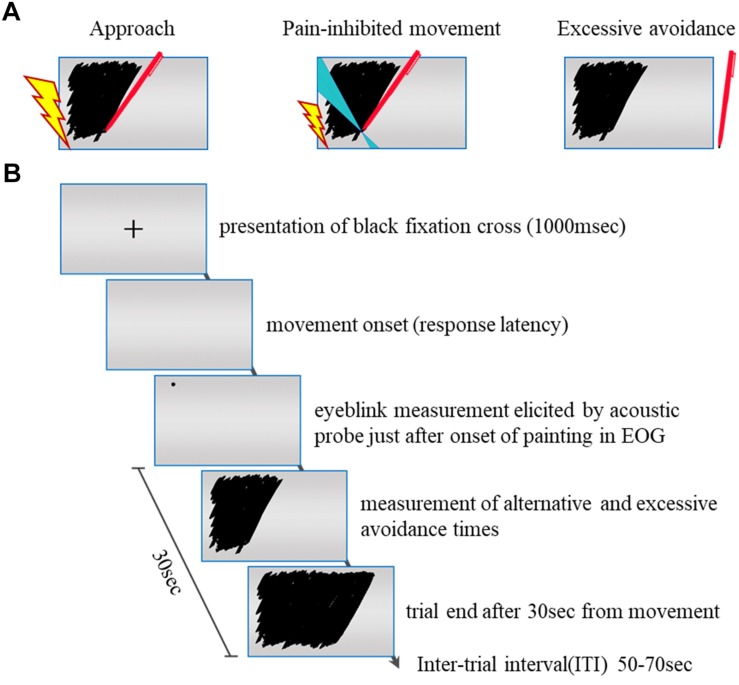
**(A)** Pain stimulation condition. We examined each subject’s painting movement (approach) with an intense painful electrocutaneous stimulus and his or her specific movement (pain-inhibited movement) with a weak painful stimulus and whether and at what time point the subject stopped painting (excessive avoidance) with no painful stimulus. The lightning bolt represents the presentation of the pain stimulus. Blue areas in condition B represent the specific directions (120–140°). **(B)** Flowchart of the experimental task. +: The fixation cross serving as the starting signal. The disappearance of the fixation cross is the movement onset. The subject could freely choose the three conditions in each trial: painting with intense pain stimulus, specific movement with weak pain stimulus, or stopping painting with no pain stimulus.

In the movement paradigm, the subject painted a rectangle that was displayed on a 10-in. touch panel (On-Lap 1002, GeChic) by using a touch pen with his or her dominant hand, and the painting motion was used. A single trial was completed 30 s after the subject started painting. As the subject painted the rectangle (referred to as “approach behavior”), a pain stimulus was administered to the subject. The subject was told that a subjective stimulus intensity of 8, which refers to a stimulus that is “significantly painful and demanding some effort to tolerate,” was the target. This pain stimulus was stopped as soon as the subject stopped painting (referred to as “excessive avoidance behavior”). In other words, the subject experienced pain while painting but did not experience pain when he or she stopped painting (see Figure 1A).

We defined “pain-inhibited movement” as the “option” behavior used in an attempt to minimize the pain intensity. The subjective stimulus intensity of painting was 5 on a numerical rating scale (NRS). For the pain-inhibited movement condition, we set the required painting movement direction at 120–140°, as this direction had shown the lowest contribution ratio of movement direction for all subjects in the confirmation experiment. For the left-handed subjects (*n* = 3), we set the painting movement direction at 30–50° reversed with reference to the *y*-axis. The specific movement velocity threshold was set at <50% of that shown by the same subject during the practice phase. Figure 1 summarizes the experimental task protocol.

### Stimulus Material

A nociceptive electrocutaneous stimulus (10-ms duration, 50 Hz) was administered by a commercial constant current stimulator (SEN-8203, Nihon Kohden, Tokyo) through two surface electrodes (34-mm diameter). The electrodes were attached to the wrist of the subject’s dominant hand. The location of the stimulation site remained the same throughout the experiment.

During the calibration procedure, each subject received electrocutaneous stimuli of gradually increasing intensity and was asked to indicate how painful the stimulus was on an NRS ranging from 0 (“I feel something but this is not painful, it is merely a sensation”) up to 10 (“This is the worst tolerable pain I can imagine”). Each subject was tested this way five times, and the intensity of the pain stimulus used in the trials was the average of these values.

### Eyeblink Startle Modulation

To verify whether pain-related fear was induced by our paradigm, we measured each subject’s auditory startle response (eyeblink startle) by measuring the electromyographic (EMG) activity. The EMG signal was digitized at 1,000 Hz from 500 ms before the onset of the auditory startle probe until 1,000 ms after the probe onset. The startle probe was a 100-dBA burst of white noise with an instantaneous rise time presented binaurally for 50 ms through headphones (MDR-XB450, Sony, Tokyo). The onset of the startle probe was set just after the first start of painting in each trial. To reduce the impedance between the skin surface and the electrode gel, the pasted part of the two electrodes that had been placed over the left lower orbital portion of the subject’s orbicularis oculi muscle (Blumental et al., 2005) was peeled and wiped with alcohol. The eyeblink component of the startle reflex in humans is reliably potentiated when the individual is confronted with a fear-induced cue or an anxiety-provoking context (Davis, 2000; Lang et al., 2000; Hamm and Weike, 2005) and reflects amygdala activation (Davis and Whalen, 2001). We measured startle responses to investigate whether the subjects’ implicit fear is affected by pain-related fear.

### Protocol

Each experiment took 90 min and had six phases: preparation, startle habituation, practice, acquisition, test, and extinction.

#### Preparation Phase

On the day before this experiment, each subject completed the short version of the Revised Temperament and Character Inventory (TCI-R) questionnaire (described below), in order to avoid fatigue caused by completing the TCI-R (which takes approximately 30 min) on the day of the experiment. On the experiment day, the subject was informed (orally and in writing) that painful electrocutaneous stimuli and loud noises would be administered during the experiment. After signing the informed consent form, the subject went to the experimental room. The subject sat on a chair, and the electrodes for the eyeblink startle responses were attached. After the stimulation electrodes were placed on the subject’s wrist, the intensity level of the pain stimulus was determined based on the results of that subject’s calibration procedure.

#### Startle Habituation Phase

In order to remove high startle responses to the first probes, the subject performed 30 trials with a startle probe (white noise, 50-ms duration) after the onset of painting. In this phase, no pain stimulus was administered; the purpose of this phase was simply to habituate the subject to the startle probe.

#### Practice Phase

The subject was instructed to paint a rectangle that was displayed on the touch panel as fully as possible within 30 s using the touch pen. This phase comprised five trials, and no pain stimulus was delivered during this phase.

#### Acquisition Phase

We investigated the acquisition process of pain-related avoidance behaviors and fear responses. Pain stimulus constantly accompanied the subject’s painting movement, and when the subject’s painting movement stopped, the pain stimulus was discontinued. This phase comprised five trials.

#### Test Phase

Only in the test phase did we add a pain-inhibited movement condition: a specific movement with decreased pain. Before the test phase, the subject received an instruction that the intensity of the pain stimulus would be reduced when the subject painted slowly to the left diagonal corner of the rectangle (in the case of left-handed subjects, the right diagonal direction was used). The subject was also told that he or she would receive a monetary reward after the experiment that was based on the size of the painted areas, and the subject was instructed to freely make decisions about whether to perform movements (i.e., approach avoidance, pain-inhibited movement, or excessive avoidance). The maximum monetary reward amount was 1,500 yen, and the amount was calculated from the average of all trials in the test phase.

The subjects were not given a chance to practice pain-inhibited movement beforehand, and thus, the subjects were supposed to perform the task in order to work toward the monetary reward with minimized pain intensity. Painting with intense pain increased the monetary reward; pain-inhibited movement with weak pain limited the monetary reward because of the slow velocity; excessive avoidance behavior with no pain did not result in any monetary reward. The subjects were instructed to freely make their own decisions regarding pain avoidance and monetary reward conflicts. This phase comprised three sets of five trials.

#### Extinction Phase

No pain stimulus was administered during the extinction phase (the same setting as that of the practice phase was used). This phase comprised five trials.

### Outcome Measures

#### Pain-Inhibited Movement or Excessive Pain-Related Avoidance Behaviors

We measured the time that a subject spent not painting as excessive avoidance behavior (the excessive avoidance time) and the time that he or she spent performing a specific movement (i.e., pain-inhibited movement time) during every trial, and the averages for each phase or set were calculated.

#### Response Latency

A fixed cross was presented in the center of the touch panel at the beginning of a trial, and its disappearance was the cue for the subject to begin movement. The movement onset latency was defined as the time from the disappearance of the cross until the touch pen contacted the touch panel. This latency indicated freezing-like guarding (Karos et al., 2015).

#### Startle Responses

The eyeblink startle response reflects a potential activation of the amygdala (Szabadi, 2012) by an intense pain stimulus. We analyzed each subject’s eyeblink EMG response using the software program MATLAB (2018b). The EMG data were digitally filtered (30–500-Hz passband) and rectified, and then we calculated the peak amplitudes (defined as the maximum of the response curve within 21–175 ms after the startle probe onset). Every peak amplitude was determined by subtracting its baseline score (the averaged EMG level between 1 and 20 ms after the probe onset). The raw scores were transformed to *z*-scores to account for interindividual differences in physiological reactivity. To optimize the visualization of the startle data and avoid negative values on the *y*-axis, we show *T*-scores (a linear transformation of the *z*-scores) in the figures. Averages were also calculated for the startle responses during painting movements.

#### The Value of Motivation to Perform Movement With Pain

We used a visual analog scale (VAS) to evaluate the extent to which the subjects felt that the movement accompanied by reward and pain had meaning or value. This scale ranged from 0 (“I do not feel that the movement had any value at all”) up to 10 (“I feel that the movement was very worthwhile”).

#### Trait Anxiety

We measured the subjects’ trait anxiety with the State–Trait–Anxiety Inventory (STAI). This scale is a well-validated, 20-item questionnaire addressing the emotional and cognitive aspects of anxiety, targeting traits of the respondent’s feelings. Each subject rated his or her feelings on a 4-point intensity scale. The total STAI score was determined by aggregating the responses to the 20 items.

#### Temperament and Character

The subjects’ temperament and character were measured with the short version of the TCI-R (Cloninger, 1994), which is a self-reported questionnaire designed to measure four aspects of temperament and three character dimensions. The TCI-R questionnaire is made up of 140 items rated on a 5-point Likert scale, ranging from 1 (definitely false) to 5 (definitely true).

### Data Analysis

We used the software program R (version 3.4.1) for all of the statistical analyses. To determine whether the subjects acquired pain-inhibited movement or excessive pain-related avoidance behavior, we performed a cluster analysis (Gaussian mixture model) of the excessive avoidance time and the pain-inhibited movement time of all three test-phase sets. We used a new experimental paradigm to calculate the excessive avoidance time and pain-inhibited movement time. However, we did not have criteria to divide the subjects into subgroups on the basis of avoidance behavior, and we thus determined and classified subjects by performing a cluster analysis. This Gaussian mixture model-based clustering with the Bayesian information criterion is used to find the most stable distribution of the mixture components. Clustering is the process of dividing a set of unlabeled data into a number of groups in such a way that samples which are similar in nature belong to the same cluster, whereas dissimilar samples are members of different clusters (Bishop, 2006).

To investigate the implications of the clusters of pain-inhibited movement and excessive avoidance times in four blocks (the acquisition phase and all three sets in the test phase), we used Kruskal–Wallis tests because of the non-normal distribution of the results of Shapiro–Wilk tests. Bonferroni correction was used to adjust the *p*-values obtained in the *post hoc* analyses. The significance level was set at *p* < 0.004.

To verify whether pain-related fear was achieved, we compared the clusters of the response latency results and the startle responses observed in the practice phase with those in five blocks (the acquisition phase, the three sets of the test phase, and the extinction phase) using the Friedman test, with Wilcoxon signed-rank tests for *post hoc* analyses. The significance level was set at *p* < 0.002 following Bonferroni correction. We analyzed the results of the subjects’ psychological evaluations (i.e., the VAS results for movement with pain, the STAI-trait scores, and the TCI-R scores) among the clusters using a series of repeated-measures one-way analyses of variance (ANOVAs; *p* < 0.05).

## Results

### Pain-Related Avoidance Behaviors

**FIGURE 2:**
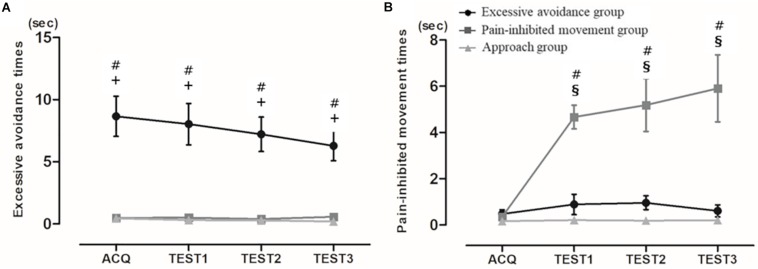
**(A)** Mean pain-inhibited movement times and **(B)** mean excessive avoidance times. Average time of specific movement or stopping movement (avoidance from pain) (mean ± SE). ACQ: acquisition phase, TEST1: first set of the test phase, TEST2: second set of the test phase, TEST3: third set. #: significant difference between clusters 1 and 2, +: significant difference between clusters 1 and 3, §: significant difference between clusters 2 and 3 (*p* < 0.004).

The third cluster showed the achieved minimum Bayesian information criterion value: first cluster, 1,375.80; second cluster, 979.97; third cluster, 614.76; fourth cluster, 631.32; and fifth cluster, 1,002.82. We divided the subjects into three subgroups based on their avoidance times: cluster 1 (*n* = 10), the “excessive avoidance group”; cluster 2 (*n* = 10), the “pain-inhibited movement group”; and cluster 3 (*n* = 24), the “approach group.” We compared the clusters to determine their meaning. Figure 2A displays the excessive avoidance times, and Figure 2B provides the pain-inhibited movement times in the acquisition phase and all test phases. Kruskal–Wallis tests of the excessive avoidance times showed significant main effects in all phases, and the excessive avoidance times of the cluster 1 subjects were significantly longer than those shown by the cluster 2 and cluster 3 subjects in all phases: acquisition phase, χ^2^ = 21.47, *p* = 0.00013; first set of the test phase, χ^2^ = 22.88, *p* < 0.0001; second set, χ^2^ = 22.84, *p* < 0.0001; third set, χ^2^ = 25.04, *p* < 0.0001.

The statistical analysis of the subjects’ pain-inhibited movement times revealed no significant differences among the three clusters in the acquisition phase and extended pain-inhibited movement times in cluster 2 of all test phases: acquisition phase, χ^2^ = 2.38, *p* = 0.30; first set of the test phase, χ^2^ = 21.87, *p* < 0.0001; second set, χ^2^ = 23.01, *p* < 0.0001; third set, χ^2^ = 22.28, *p* < 0.0001. In other words, this result showed that the subjects in cluster 2 made a clear decision to perform pain-inhibited movement.

### Response Latencies

**FIGURE 3:**
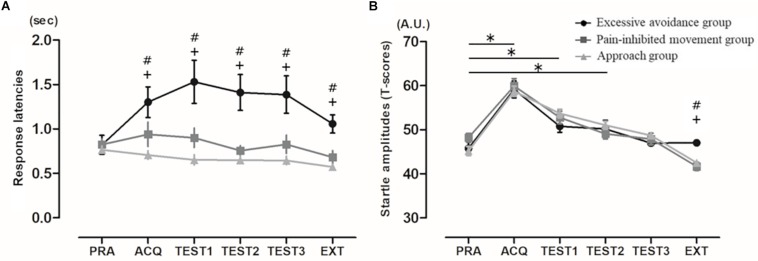
**(A)** Mean response latencies (mean ± SE). EXT: extinction phase. #: significant difference between clusters 1 and 2, +: significant difference between clusters 1 and 3 (*p* < 0.003). **(B)** Mean startle amplitudes. Mean eyeblink startle amplitudes (mean ± SE). For graphic purposes, *T*-scores were used. PRA: practice phase. ^∗^significant difference between practice phase and other phases, #significant difference between clusters 1 and 2, +: significant difference between clusters 1 and 3.

As shown in Figure 3A, in the comparisons of the mean response latencies for the six blocks, the Friedman test showed a significant main effect (χ^2^ = 18.25, *p* < 0.005). However, the *post hoc* test showed that the subjects’ response latencies during the acquisition, test, and extinction phases have no significant effects compared to those in the practice phase: acquisition phase, *p* = 1.00; first set of the test phase, *p* = 1.00; second set, *p* = 1.00, third set, *p* = 1.00; and extinction phase, *p* = 0.18. In addition, the mean response latencies of the cluster 1 subjects were significantly longer than those shown by the cluster 3 subjects (but not the cluster 2 subjects) in the acquisition phase and were significantly delayed compared to those of the cluster 2 and 3 subjects in all test phases and the extinction phase: practice phase, χ^2^ = 1.94, *p* = 0.38; acquisition phase, χ^2^ = 16.69, *p* < 0.0001; first set of the test phase, χ^2^ = 20.45, *p* < 0.0001; second set, χ^2^ = 22.56, *p* < 0.0001; third set, χ^2^ = 21.77, *p* < 0.0001; and extinction phase, χ^2^ = 16.67, *p* = 0.0002.

### Startle Responses

In the comparisons of the startle response for the six blocks, the Friedman test showed a significant main effect (χ^2^ = 114.82, *p* < 0.0001) (Figure 3B). The *post hoc* test showed that the subjects’ startle responses during the acquisition phase and the first and second sets of the test phase were significantly higher than those in the practice phase: acquisition phase, *p* < 0.0001; first set of the test phase, *p* < 0.0001; second set, *p* = 0.001; third set, *p* = 0.066; and extinction phase, *p* = 0.004. In addition, only in the extinction phase were the startle responses of the cluster 1 subjects significantly higher than those of clusters 2 and 3 (practice phase, χ^2^ = 4.08, *p* = 0.13; acquisition phase, χ^2^ = 0.49, *p* < 0.78; first set of the test phase, χ^2^ = 3.23, *p* = 0.20; second set, χ^2^ = 1.30, *p* = 0.52; third set, χ^2^ = 2.50, *p* = 0.29; and extinction phase, χ^2^ = 17.24, *p* = 0.0002).

### Psychological Scales

**FIGURE 4:**
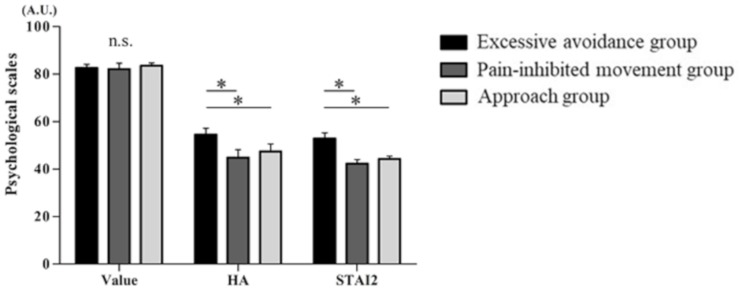
Psychological scale. From the left, the values are as follows: the visual analog scale (VAS) scores reflecting the value of movement with pain, the State–Trait–Anxiety Inventory (STAI)-trait scores, and the harm avoidance (HA) scores of the Revised Temperament and Character Inventory (TCI-R) (mean ± SE). ^∗^*p* < 0.05.

Figure 4 displays the psychological scales for each cluster of subjects. There were no significant differences among the three clusters in the VAS results for movement with pain. The STAI-trait and harm avoidance scores of the TCI-R shown by the cluster 1 subjects were significantly higher than those shown by the cluster 2 and cluster 3 subjects [VAS of the value of movement with pain, *F*(2,41) = 0.051, *p* < 0.01; STAI-trait, *F*(2,41) = 9.101, *p* < 0.01; and harm avoidance, *F*(2,41) = 6.939, *p* < 0.01].

## Discussion

We attempted to clarify the respective characteristics of the individuals who acquire avoidance behaviors and whether those behaviors protect from the extinction of fear responses. We hypothesized that the subjects who exhibit excessive avoidance have high trait anxiety and harm avoidance as personality traits and show resistance to extinction compared to other subjects. We succeeded in extracting the characteristics of healthy subjects who exhibited pain-inhibited movement and excessive pain-related avoidance behaviors by using a novel voluntary movement paradigm and cluster analysis. In this movement paradigm, the subjects painted as much as possible to obtain a monetary reward based on their own free movement decisions. They engaged in excessive avoidance behaviors, although they knew that movement in a specific direction (i.e., pain-inhibited movement) could decrease the pain intensity. The summary of results per cluster was described in Table 1.

**TABLE 1:** Summary of results per cluster.

		**Excessive avoidance**		**Pain-inhibited movement**		**Approach**		**Effect size**
		**group (*n* = 10)**		**group (*n* = 10)**		**group (*n* = 24)**			
		**Mean**	**SE**	**Mean**	**SE**	**Mean**	**SE**	**η^2^**
Excessive avoidance times (sec)	ACQ	8.66	1.61	0.49	0.19	0.45	0.16	0.67
	TEST1	8.03	1.66	0.51	0.16	0.31	0.06	0.64
	TEST2	7.22	1.39	0.39	0.13	0.29	0.07	0.67
	TEST3	6.29	1.20	0.58	0.17	0.20	0.04	0.67
Pain-inhibited movement times (sec)	ACQ	0.48	0.18	0.35	0.12	0.17	0.04	0.12
	TEST1	0.89	0.44	4.67	0.51	0.21	0.03	0.78
	TEST2	0.96	0.30	5.19	1.14	0.19	0.03	0.59
	TEST3	0.61	0.26	5.91	1.45	0.20	0.04	0.55
Response latencies (sec)	PRA	0.82	0.11	0.83	0.06	0.77	0.04	0.17
	ACQ	1.30	0.17	0.94	0.14	0.71	0.05	0.49
	TEST1	1.53	0.24	0.90	0.11	0.65	0.06	0.59
	TEST2	1.41	0.20	0.76	0.05	0.65	0.04	0.69
	TEST3	1.39	0.21	0.83	0.10	0.65	0.05	0.59
	EXT	1.06	0.10	0.68	0.07	0.57	0.04	0.69
Startle responses (A.U.)	PRA	45.81	1.39	48.23	0.99	45.20	1.03	0.09
	ACQ	59.17	1.89	59.96	1.65	58.76	1.14	0.00
	TEST1	50.78	1.35	52.81	1.38	53.71	0.94	0.07
	TEST2	50.22	1.96	49.08	1.15	51.04	0.91	0.03
	TEST3	46.98	0.76	47.92	1.32	48.73	0.80	0.03
	EXT	47.04	0.85	41.70	0.90	42.55	0.41	0.43
Value of motivation		82.60	1.49	82.10	2.51	83.54	1.17	0.00
STAI-trait		52.90	2.35	42.40	1.61	44.25	1.20	0.31
Harm avoidance		54.50	2.68	44.90	3.28	47.46	3.05	0.25

*Mean, standard error (SE), and effect size in the intergroup comparisons per cluster.*

Interestingly, the subjects who exhibited excessive avoidance behavior delayed the onset of painting movement, and their startle responses remained in the extinction phase, during which no pain stimulus was administered. Additionally, these subjects had higher trait anxiety and high harm avoidance scores on the TCI-R. These results were generally consistent with our hypothesis.

We used a cluster analysis to classify the 44 subjects’ pain-related behavioral traits and divided them into three groups on the basis of their avoidance times: the cluster 1 subjects, the “excessive avoidance group:; the cluster 2 subjects, that is, the “pain-inhibited movement group”; and the cluster 3 “approach group.” These behaviors were retained through the acquisition and test phases. We observed that the cluster 1 (excessive avoidance) subjects hesitated to initiate movement. The response latencies of the cluster 1 subjects were delayed compared to those of the cluster 2 and cluster 3 subjects, not only in the test phase but also in the extinction phase, indicating that freezing-like guarding persisted despite the pain-free condition (Karos et al., 2015). We suggest that such residual guarding behavior occurred because the subjects’ learned excessive avoidance persisted. In relation to this, individuals with complex regional pain syndrome (CRPS) suffering from severe pain had delayed movement onset (Schilder et al., 2012; Christophe et al., 2016). Our present results suggest that the delay of the start of CRPS movement as reported in the past may be caused by learning the “fear of pain.”

We also observed that the subjects’ startle responses were higher in the acquisition phase and the first test phase compared to the practice phase, which indicates that pain-related fear was induced by the movement paradigm. Interestingly, in the extinction phase, the cluster 1 subjects showed greater physiological fear responses compared to the cluster 2 and cluster 3 subjects; that is, the fear response remained in the excessive avoidance group regardless of the painless condition. This physiological phenomenon is an important and interesting result that supports results of not canceling out pain-related fear in people who show excessive avoidance behaviors and movement onset delay (Lovibond et al., 2000; Volders et al., 2015), even during an extinction phase. In addition, total duration of shock and startle responses of acquisition and test phases have no significant correlation. On the other hand, a negative correlation was suggested between total duration of shock and startle responses of extinction phase. Total duration of pain-inhibited movement have no significant correlation of startle responses in any phases (Supplementary Figure S3).

In individuals with chronic pain, fear responses remain in the extinction phase, and the persistence of excessive protective responses may contribute to the maintenance of long-term chronic pain disability (Meulders et al., 2017). This is similar to our present observation of the fear responses. In other words, pain-related movements and pain-related fear are sufficiently related in the acquisition and test phases, and pain-related fear seems to play an important role in excessive avoidance. We thus speculate that the subjects in our excessive avoidance group and individuals who avoid moving a body part in response to a physical injury share a common avoidance process that is based on pain-related fear.

In addition, habitual responses have been shown to be less sensitive to extinction (Yin and Knowlton, 2006). In other words, individuals who engage in excessive avoidance behaviors may exhibit a pattern of repeated maladaptive avoidance behaviors and may become accustomed to avoidance. Moreover, because thinking about the pain-inducing behavior induces an excessive fear of taking action, they do not move, and a vicious circle of chronic pain is created. The relationship between excessive avoidance and resistance to extinction may explain in part the phenomenon in which CRPS patients show inactivity and disuse of affected body parts, leading to chronic pain. In contrast, individuals who engage in pain-inhibited movement have no resistance to extinction, although they have a habit of avoiding pain.

Active avoidance is known to involve the attenuation of conditioned responses mediated by the amygdala, ventromedial prefrontal cortex (VmPFC), and striatum (LeDoux, 2000; Cain and LeDoux, 2007; Bravo-Rivera et al., 2015; Maier, 2015; Boeke et al., 2017). The striatum responds to prediction errors which lead to avoidance due to fear (Seymour et al., 2005, 2012; Schiller et al., 2008; Delgado et al., 2009; Collins et al., 2014; Eldar et al., 2016), and VmPFC activation by active avoidance learning inhibits the function of the amygdala (Baratta et al., 2007; Moscarello and LeDoux, 2013; Ramirez et al., 2015). Thus, pain-inhibited movement, which is a proactive coping strategy, inhibits delayed movement onset and is an adaptive response to pain-related fear. In clinical situations, active movements such as pain-inhibited movement may mitigate the chronicity of pain.

Pain is one of the inducers of avoidance, but occasionally, there is a conflict when an individual must engage in painful behaviors in social life, and in the present study, the painful behavior was a confrontational task with a target orientation of a monetary reward. In such an approach–avoidance competition, the value to be gained by performing the task is an important factor in the individual’s decision making, but we observed that this value was irrelevant to any behaviors in the present experiment. If the subjects had been offered no reward in this study, the number of subjects who avoided the painful stimulus might have been larger. Despite feeling a sense that the approach behavior would have value, some subjects showed excessive avoidance behavior. Those subjects also had high trait anxiety and showed high harm avoidance. People with anxiety disorder (Dymond and Roche, 2009; Meulders et al., 2014; Sheynin et al., 2014) and those with high harm avoidance (Paulus et al., 2003; Markett et al., 2016) are known to avoid feared events immediately, and the same tendency was also observed with regard to the painful event in the present experiments.

Some study limitations should be addressed. First, we conducted the extinction phase as only one set of trials in order to prevent fatigue in the subjects due to an extension of the experimental time and the problems that could cause. The subjects engaged in approach behavior with no pain for 30 s in this phase. It is an important finding that the subjects who showed excessive avoidance behavior continued to exhibit fear responses, whereas the other subjects showed diminished fear responses in the extinction phase of this experimental protocol. Second, psychological traits could not be identified in the pain-inhibited movement group in the comparison of this group with the approach group. This may be a limitation of immediate conditioning in healthy subjects, because patients with low back pain who have high levels of fear acquire pain-inhibited movement (Thomas and France, 2008). Third, the experimental paradigm, while interesting and internally valid, has significant limitations as a model of clinical pain and pain avoidance. It is not clear whether the consequences of avoiding a painful experimental stimulus are anything like the consequences associated with clinical pain, in intensity or in quality. Fourth, the maximum reward that subjects could gain was 1,500 yen. We evaluated the importance of motivating subjects to perform movements that induced pain, and all of the subjects reported that the motivation was valuable in prompting them to perform the approach behavior. We consider that balance with mixed approach–avoidance task in this study may be sufficient in a characteristic of recruitment. Fourth, we did not set up a control group for comparison in the test phase; all groups are treated identically and then divided into groups based on performance. Since the purpose of this study was to clarify the behavioral differences in movement-related pain, a protocol was created with an emphasis on clarifying the results and conclusions without setting up a control group. However, a control group would be useful for gaining a better understanding of pain-related behaviors.

## Conclusion

In conclusion, although all of the subgroups of subjects showed fear due to pain in this study, they showed different pain-related avoidance behaviors and different conceptualizations of pain behavior. Consequently, their excessive pain-related avoidance behaviors were related to the subgroups’ personality traits. We suggest that when evaluating an individual’s pain in clinical practice, it is important to evaluate aspects of the individual’s temperament and his or her past experiences and thinking. This knowledge informs us of the importance of evaluating avoidance behaviors in detail to prevent development or maintenance of chronic pain.

## Data Availability Statement

All datasets generated for this study are included in the manuscript/Supplementary Files.

## Ethics Statement

The studies involving human participants were reviewed and approved by Akimichi Kaneko Kio University. The patients/participants provided their written informed consent to participate in this study.

## Author Contributions

YN conceived and designed the study, and acquired, analyzed, and interpreted the data. MO conceived and designed the study, and critically revised the manuscript for important intellectual content. SN conceived and designed the study. KT analyzed the data. SM supervised the study.

## Conflict of Interest

The authors declare that the research was conducted in the absence of any commercial or financial relationships that could be construed as a potential conflict of interest.

## Supplementary Material

FIGURE S1 **(A)** Pain stimulation condition. Painting (approach) with the painful electrocutaneous stimulus and stopping painting (avoidance) with no painful stimulus. The lightning bolt represents the presentation of the pain stimulus. **(B)** Flowchart of the confirmation experimental task. The acoustic probe was administered after the onset of arbitrary movement measured by an electro-oculogram (EOG). In the trial, the subject could freely decide whether to continue painting with the pain stimulus or to stop painting with no pain stimulus.

FIGURE S2 **(A)** Mean avoidance time. The average time of stopping movement (avoidance from pain) (mean ± SE). ACQ: acquisition phase, TEST1: first set of test phase, TEST2: second set, TEST3: third set, TEST4: fourth set of the test phase. ^#^*p* < 0.01, cluster 1 vs. cluster 2. Based on the results of the cluster analysis (Ward’s method), the subjects could be divided into the following two subgroups: cluster 1 (*n* = 6) and cluster 2 (*n* = 18). We compared the clusters and observed that the avoidance times of cluster 1 subjects were significantly longer than those of cluster 2 subjects in all phases (acquisition phase, *p* = 0.0009; first set of the test phase, *p* = 0.0002; second set, *p* = 0.0006; third set, *p* = 0.0004; and fourth set, *p* = 0.007). **(B)** Mean startle amplitudes. Mean eyeblink startle amplitudes (mean ± SE). Note that for graphic purposes, *T*-scores were used. PRA: practice phase. ^∗^*p* < 0.0045, practice phase vs. the other phases. In our comparison of the startle responses in six blocks, the Friedman test showed a significant main effect (χ^2^ = 53.88, *p* < 0.001). The *post hoc* test showed that for all 24 subjects, the startle response in the acquisition phase was significantly higher than that in the practice phase (*p* < 0.001) but the startle response for all test phases was not significantly different from that of the practice phase (first set of the test phase, *p* = 0.002; second set, *p* = 0.72; third set, *p* = 0.79; fourth set, *p* = 0.08). Moreover, in all phases or sets, there were no significant differences between the two clusters.

FIGURE S3 **(A)** The correlation between the total times of pain-inhibited movements and the mean startle amplitudes (*T*-scores) of the ACQ and TEST phases. **(B)** The correlation between the total times of approach movements and the mean startle amplitudes (*T*-scores) of the ACQ and TEST phases. **(C)** The correlation between the total times of pain-inhibited movements and the mean startle amplitudes (*T*-scores) of the EXT phase. **(D)** The correlation between the total times of approach movements and the mean startle amplitudes (*T*-scores) of the EXT phase. Pearson’s correlation coefficient was used for the statistical analysis. ACQ, acquisition; TEST, test; EXT, extinction; *r*, correlation coefficient; *p*, *p*-value.
