# Supplementary figures and images for: Avoidance Behavioral Difference in Acquisition and Extinction of Pain-Related Fear
Source: Front Behav Neurosci. 2019 Oct 11;13:236. doi: 10.3389/fnbeh.2019.00236 (PMC6797557; doi:10.3389/fnbeh.2019.00236)

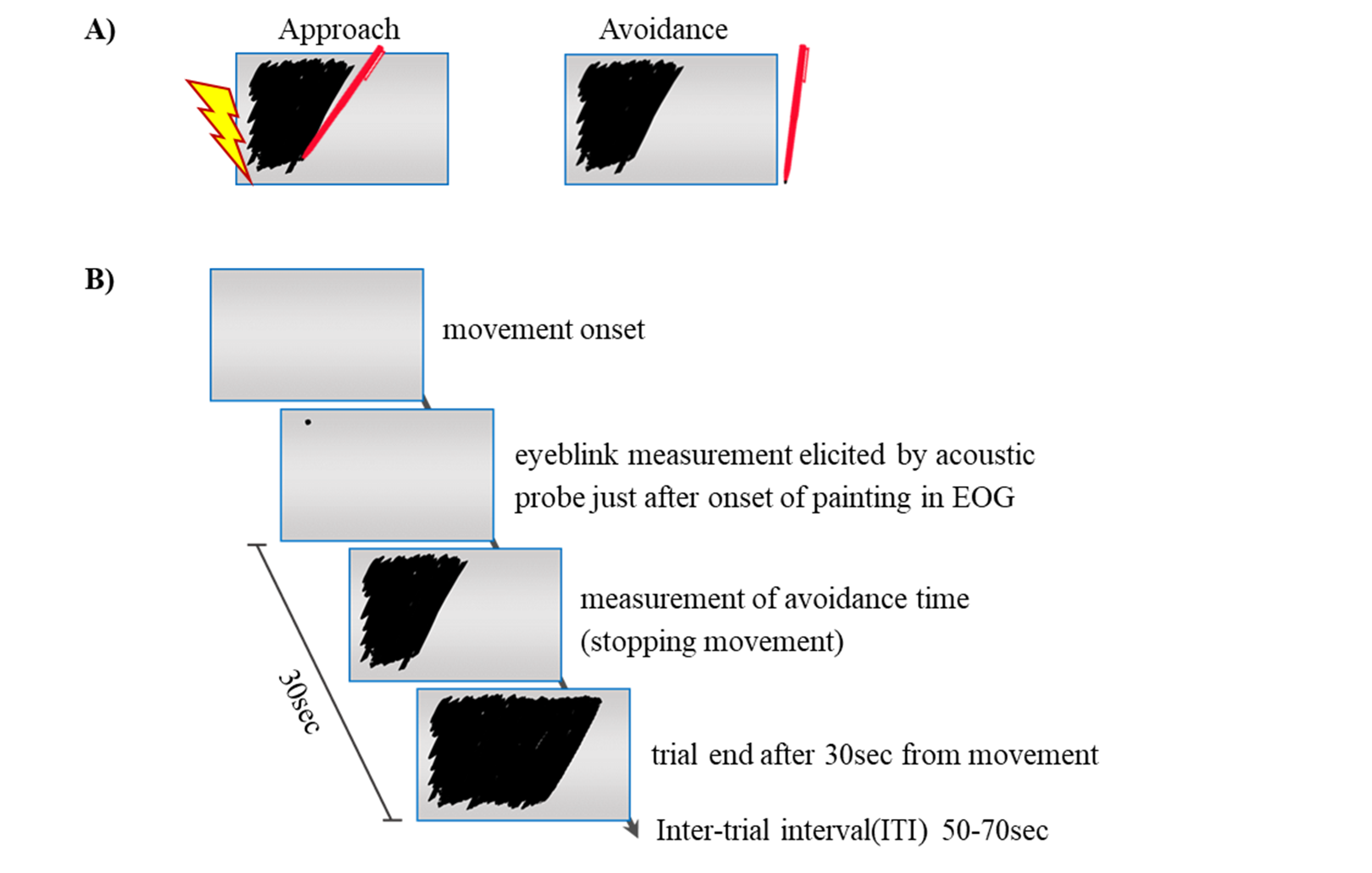

Supplement: FIGURE S1 — (A) Pain stimulation condition. Painting (approach) with the painful electrocutaneous stimulus and stopping painting (avoidance) with no painful stimulus. The lightning bolt represents the presentation of the pain stimulus. (B) Flowchart of the confirmation experimental task. The acoustic probe was administered after the onset of arbitrary movement measured by an electro-oculogram (EOG). In the trial, the subject could freely decide whether to continue painting with the pain stimulus or to stop painting with no pain stimulus. [file Image_1.TIF]

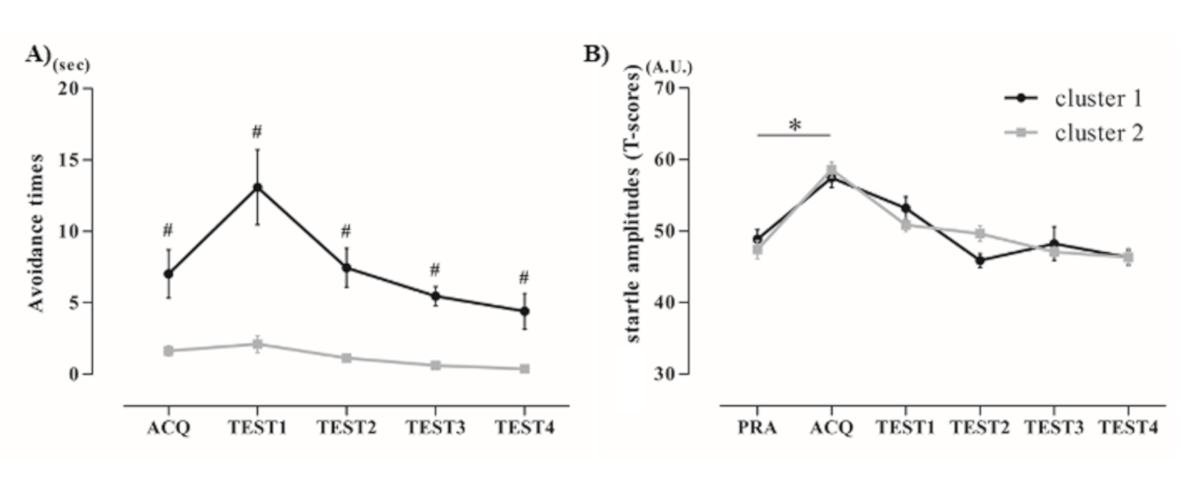

Supplement: FIGURE S2 — (A) Mean avoidance time. The average time of stopping movement (avoidance from pain) (mean ± SE). ACQ: acquisition phase, TEST1: first set of test phase, TEST2: second set, TEST3: third set, TEST4: fourth set of the test phase. #p < 0.01, cluster 1 vs. cluster 2. Based on the results of the cluster analysis (Ward’s method), the subjects could be divided into the following two subgroups: cluster 1 (n = 6) and cluster 2 (n = 18). We compared the clusters and observed that the avoidance times of cluster 1 subjects were significantly longer than those of cluster 2 subjects in all phases (acquisition phase, p = 0.0009; first set of the test phase, p = 0.0002; second set, p = 0.0006; third set, p = 0.0004; and fourth set, p = 0.007). (B) Mean startle amplitudes. Mean eyeblink startle amplitudes (mean ± SE). Note that for graphic purposes, T-scores were used. PRA: practice phase. ∗p < 0.0045, practice phase vs. the other phases. In our comparison of the startle responses in six blocks, the Friedman test showed a significant main effect (χ2 = 53.88, p < 0.001). The post hoc test showed that for all 24 subjects, the startle response in the acquisition phase was significantly higher than that in the practice phase (p < 0.001) but the startle response for all test phases was not significantly different from that of the practice phase (first set of the test phase, p = 0.002; second set, p = 0.72; third set, p = 0.79; fourth set, p = 0.08). Moreover, in all phases or sets, there were no significant differences between the two clusters. [file Image_2.tif]

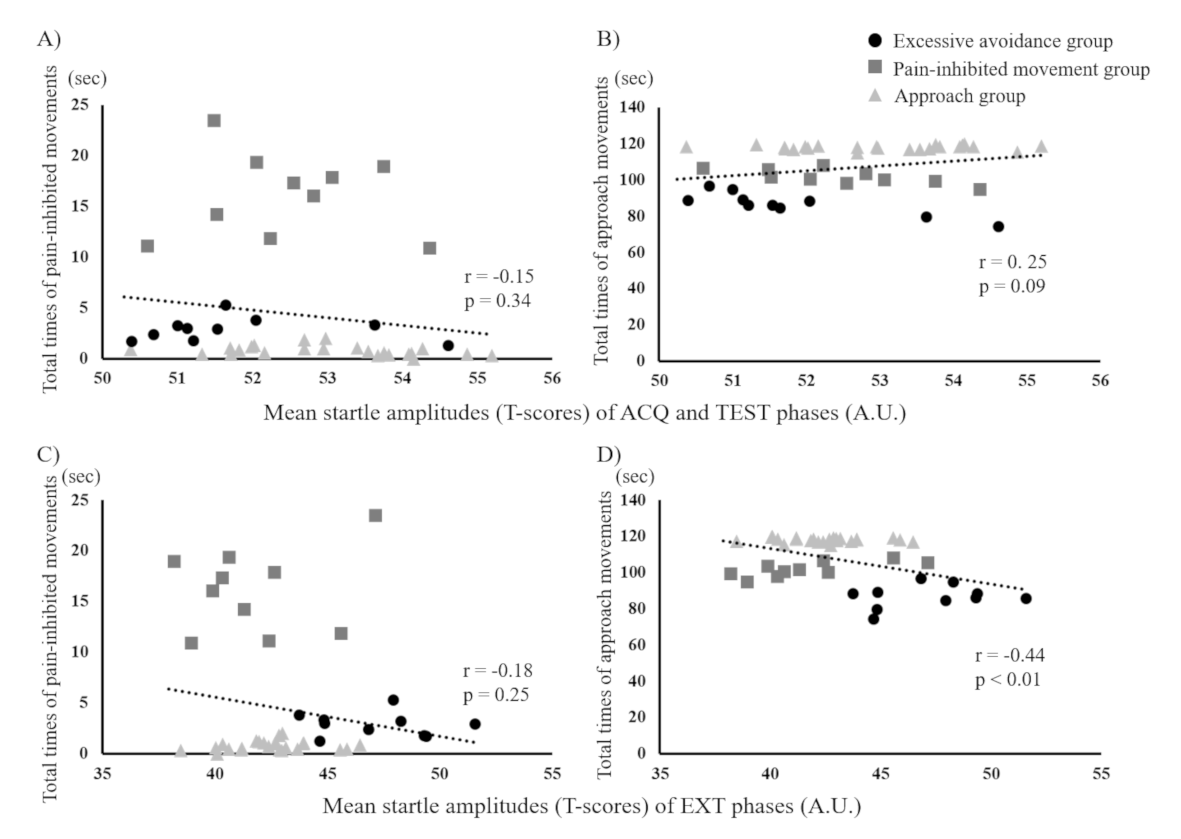

Supplement: FIGURE S3 — (A) The correlation between the total times of pain-inhibited movements and the mean startle amplitudes (T-scores) of the ACQ and TEST phases. (B) The correlation between the total times of approach movements and the mean startle amplitudes (T-scores) of the ACQ and TEST phases. (C) The correlation between the total times of pain-inhibited movements and the mean startle amplitudes (T-scores) of the EXT phase. (D) The correlation between the total times of approach movements and the mean startle amplitudes (T-scores) of the EXT phase. Pearson’s correlation coefficient was used for the statistical analysis. ACQ, acquisition; TEST, test; EXT, extinction; r, correlation coefficient; p, p-value. [file Image_3.tif]
